# Chairside Fabrication of an All-Ceramic Partial Crown Using a Zirconia-Reinforced Lithium Silicate Ceramic

**DOI:** 10.1155/2016/1354186

**Published:** 2016-03-06

**Authors:** Sven Rinke, Anne-Kathrin Pabel, Matthias Rödiger, Dirk Ziebolz

**Affiliations:** ^1^Dental Practice, 63456 Hanau, Germany; ^2^Department of Prosthetics, University Medical Center Göttingen, 37075 Göttingen, Germany; ^3^Department of Cariology, Endodontology and Periodontology, University of Leipzig, 04103 Leipzig, Germany

## Abstract

The chairside fabrication of a monolithic partial crown using a zirconia-reinforced lithium silicate (ZLS) ceramic is described. The fully digitized model-free workflow in a dental practice is possible due to the use of a powder-free intraoral scanner and the computer-aided design/computer-assisted manufacturing (CAD/CAM) of the restorations. The innovative ZLS material offers a singular combination of fracture strength (>370 Mpa), optimum polishing characteristics, and excellent optical properties. Therefore, this ceramic is an interesting alternative material for monolithic restorations produced in a digital workflow.

## 1. Introduction

Providing a tooth-colored restoration in only one appointment is the main goal of the chairside concept with computer-aided design/computer-assisted manufacturing (CAD/CAM) technology, which was first realized with the introduction of the CEREC-system [1]. The CEREC-system evolved through a combination of numerous software and hardware upgrades since its launch more than 30 years ago [2]. Meanwhile, the CAD/CAM of dental restorations has become an established fabrication process, especially for all-ceramic solutions [1–4].

Long-term survival rates for CAD/CAM-fabricated inlays and onlays appear to be similar to traditional restorations [5]. The survival possibility of CEREC-generated restorations fabricated from machinable feldspathic porcelain (Vita MK II, Vita Zahnfabrik, Bad Säckingen, Germany) was reported to be approximately 97% for 5 years and 90% for 10 years [5, 6]. For observational periods of up to 17 years, a survival rate of 88.7% was calculated [7]. The most frequent reason for failure was a ceramic fracture (62%) followed by tooth fractures (14%) and caries (19%). To overcome this limitation, new materials with improved fracture strength (e.g., lithium disilicate ceramics (Ls2)) >350 MPa were introduced for the manufacturing of extended CAD/CAM restorations (partial crowns and full coverage crowns) in the posterior region [8]. Reich and Schierz (2013) assessed the clinical performance of chairside-generated lithium disilicate crowns during an observational period of 4 years [9]. The failure-free rate was 96.3% after 4 years according to Kaplan-Meier analysis. This is comparable with the survival rates reported for posterior metal-ceramic crowns and conventional heat-pressed crowns made from lithium disilicate ceramics [10].

Recent material-related research focuses on the development of CAD/CAM materials offering improved mechanical strength, combined with adequate translucency, and time-saving fabrication [11]. Recently, a new group of machinable ceramics for CAD/CAM techniques has been launched: zirconia-reinforced lithium silicate (ZLS) ceramics (Celtra Duo, Dentsply DeTrey, Konstanz, Germany; Suprinity, Vita Zahnfabrik, Bad Säckingen, Germany). Both materials are supported by CEREC version 4.2 and above. Pursuant to the manufacturers, the mechanical properties of these materials range between 370 and 420 MPa. The addition of 8–10 wt% zirconium oxide leads to an improved strength [12]. After crystallization, the material has a homogeneous texture (mean grit size: approximately 0.5–0.7 *μ*m). The formed crystals are 4 to 8 times smaller than lithium disilicate crystallites. ZLS-ceramics consist of a dual microstructure: the first component is very fine lithium metasilicate with lithium disilicate crystals (average size: 0.5–0.7 *μ*m). This is the main difference from Ls2 ceramics, which only contain lithium disilicate crystals. The second component is the glassy matrix containing 10% zirconium oxide in solution. The result is a very fine microstructure that allows a high flexural strength while at the same time providing a high percentage of glassy matrix, thus leading to good optical, milling, and polishing properties [12].

Currently, the ZLS-ceramics are sold as Suprinity (Vita Zahnfabrik) and Celtra Duo (Dentsply DeTrey) for chairside as well as lab site processing. The zirconia-reinforced silicate ceramic Vita Suprinity is a precrystallized ceramic material. Accordingly, the CAM processing is comparable with lithium disilicate ceramic materials (crystallization firing after milling to achieve the final density). However, the ZLS variation Celtra Duo (Dentsply DeTrey) is a finally crystallized ceramic. This is especially suitable for chairside application, as the final workpiece is available after a milling time of only 10 to 22 minutes. The milled restorations have a flexural strength of 210 MPa. An additional stain and glaze firing will increase the material's flexural strength to 370 MPa [13]. Thus, the final crystallized ZLS variations offer an up-to-date combination of short processing times and high stability. These properties especially allow the chairside fabrication of all-ceramic restorations in the posterior region.

Meanwhile, the mechanical, bonding, and optical properties of these final crystallized ZLS-ceramics (i.e., Celtra Duo) were tested in comparative university-based in vitro studies [13–16]. These tests revealed a fracture strength and a marginal adaptation comparable to the clinically well-proven lithium disilicate (Ls2) glass ceramics [13]. Moreover, under in vitro conditions, the wear and volumetric loss for the glaze-fired ZLS-ceramic Celtra Duo was not significantly different from human enamel [15]. The in vitro evaluation of the bonding properties showed an encouraging bonding performance of the Celtra Duo crowns when pretreated as recommended by the manufacturer (hydrofluoric acid etching), being sufficient to withstand intraoral chewing forces during mastication [16].

This case report describes a treatment with monolithic ceramic restorations which are fabricated from ZLS-ceramics (Celtra Duo Dentsply DeTrey, Konstanz, Germany), in a fully digitized workflow with a powder-free intraoral scanner (CEREC Omnicam, Sirona Bensheim, Germany) and a practice-based milling system (CEREC MCXL, Sirona, Bensheim, Germany).

## 2. Case Presentation

**Figure 1:**
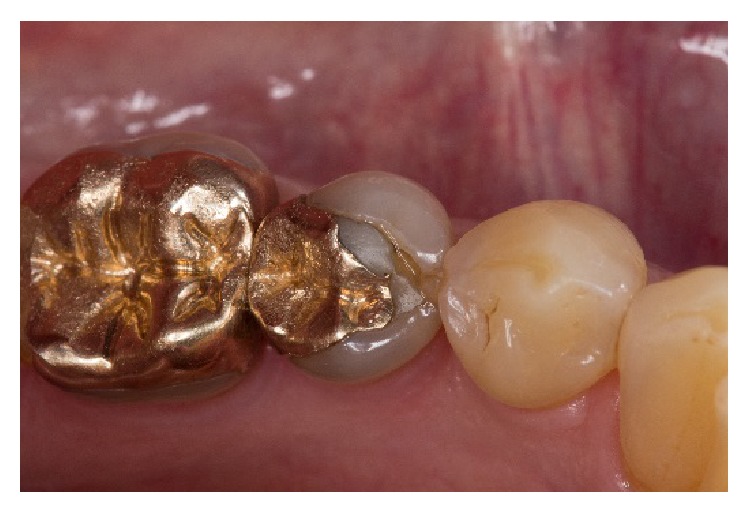
Insufficient cast gold inlay on a lower second premolar.

A 62-year-old man presented with a need for restorative treatment of the lower left second premolar. The existing cast gold inlay showed an insufficient fitting accuracy, and repeated clinical intervention had been necessary due to loss of retention (Figure 1). The tooth was vital; a systematic periodontal treatment led to a stable overall periodontal situation. The patient wanted a replacement of the cast gold restoration with a chairside fabricated all-ceramic partial crown. For the fabrication process of the monolithic partial crown with the CEREC-system, a fully crystallized ZLS-ceramic (Celtra Duo, Dentsply DeTrey, Konstanz, Germany) was used.

**Figure 2:**
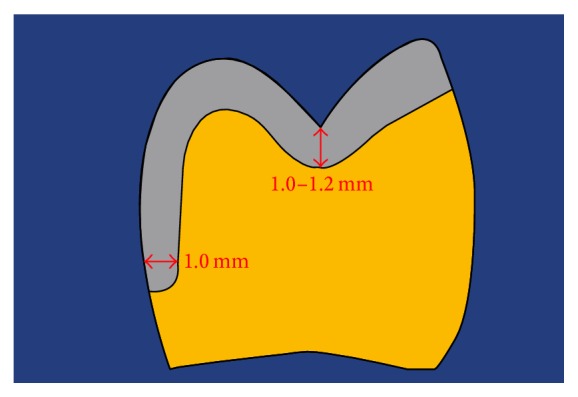
Minimum material thickness recommended for all-ceramic partial crowns fabricated from zirconia-reinforced lithium silicate (ZLS) ceramics.

At the beginning of the second clinical appointment, shade selection using a conventional shade guide (Vitapan classic, Vita Zahnfabrik, Bad Säckingen, Germany) was performed. After the application of local anesthetics (Sopira Heraeus Kulzer, Hanau, Germany), the existing inlay restoration was detached, and the caries were removed. A core build-up was placed adhesively (Core-Up OptiMix, Kaniedenta, Herford, Germany). Due to the large defect and the thin remaining buccal wall (thickness < 1.5 mm), the tooth needed a ceramic partial crown. Preparation was performed according to the recommendations of Ahlers et al. (2009) [17], avoiding sharp edges. A minimum material thickness of 1.2 to 1.0 mm in the occlusal area was maintained. The preparation limit was carried out as a shoulder preparation with internal rounded line angle and a cutting depth of 1.0 mm (Figure 2).

**Figure 3:**
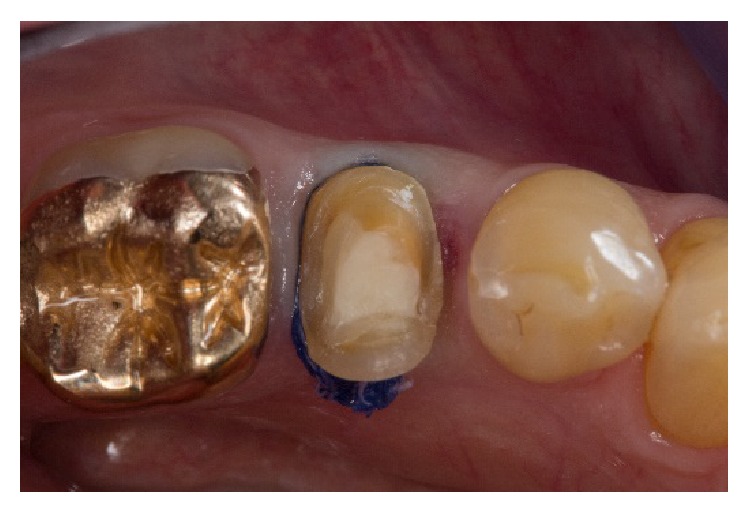
Finished preparation with retraction cords placed.

Prior to digital impression taking, two layers of nonimpregnated retraction cords (sizes 00 and 1) (UltraPak, Ultradent Products, Cologne, Germany) were placed. A V-shaped pack was created by putting a second cord with a larger diameter directly over the first, thus providing a physical lateral displacement of the tissues (Figure 3). The retraction cords were left in the sulcus while the tooth was air-dried. Due to the mainly supragingival preparation, no further preparation steps were necessary to take the digital impression. Intraoral scanning was performed with powder-free technology by using a CEREC Omnicam (CEREC Omnicam Sirona Bensheim, Germany). The CEREC Omnicam is a true-color high-resolution 3D intraoral camera using white LEDs as a light source. The 3D calculations were based on triangulation measurement.

**Figure 4:**
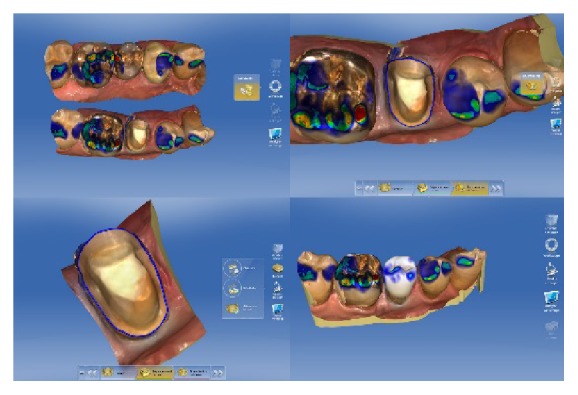
Intraoral scans with marked preparation line and design suggestion generated with the CEREC software (version 4.2).

First, the lower left quadrant of the mandible was scanned and then data in the upper left quadrant were collected. When the patient closed into an intercuspal position, a buccal scan was taken. The system implemented the digital registration to create a 3D occlusion relation (Figure 4).

**Figure 5:**
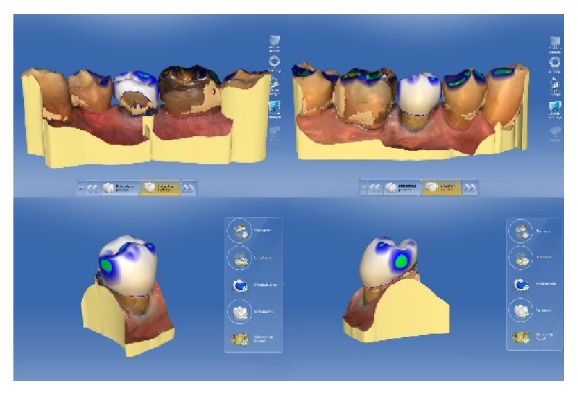
Adjustment of the occlusal and proximal contacts.

In the next step, the virtual models were adjusted, and the preparation limit was marked and edited. After the determination of the insertion path and the model axis, the automated design of the restoration was started (Figure 4). For the design of the partial crowns, the design feature “Biogeneric Individual” was selected. For this design suggestion made by the software, the neighboring teeth are analyzed, and the program extrapolates the naturally created morphology for the design of the restoration. Proximal and occlusal contact strength was set to 25 *μ*m. Spacer thickness was reduced to 50 *μ*m, and the minimum occlusal thickness was adjusted to the material specific value (1000 *μ*m). Finally, the design suggestion was slightly modified regarding position and size with the respective design tool. Furthermore, the proximal contacts were slightly enlarged (Figure 5). Based on the mainly automated design of the biogeneric feature, the time needed for the design process can be shortened to 5 minutes or less for a single-tooth restoration.

**Figure 6:**
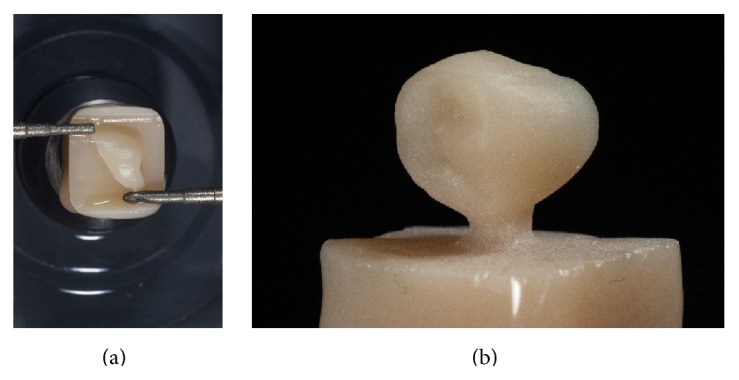
Wet grinding process of the partial crown using the CEREC MXCL (Sirona Bensheim, Germany) unit.

The restoration was then milled as a full-contour monolithic partial crown from a finally crystalized ZLS-ceramic using a practice-based compact milling unit (CEREC MCXL, Sirona, Bensheim, Germany) in a wet grinding process (Figures 6(a) and 6(b)).

The Celtra Duo material type HT (high translucency) in Vita shade A3 was selected to create a pronounced chameleon effect when milling the partial crown. Depending on the size of the restoration, the milling process takes between 10 and 14 minutes.

After machining the restorations, first, the fixation bar of the restoration was removed with water-cooled diamond instruments. Then, the occlusal surfaces were reworked using the same fine-grit size instrument (8390.314.016, Gebr. Brasseler, Lemgo, Germany).

**Figure 7:**
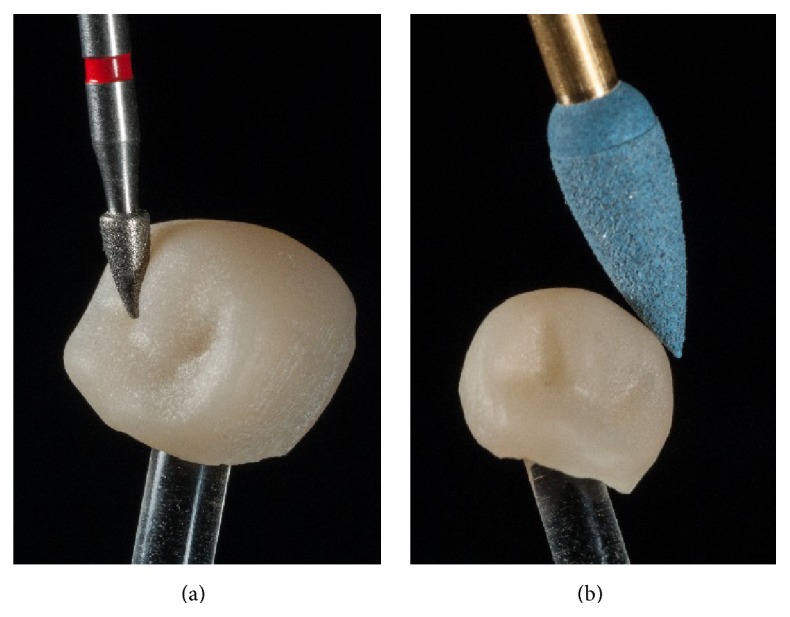
Postprocessing of the occlusal surface and prepolishing using a diamond-impregnated polyurethane polisher.

Afterwards, the complete restoration was carefully prepolished with diamond-impregnated polyurethane instruments (94020C.204.040. Gebr. Brasseler, Lemgo, Germany) (Figures 7(a) and 7(b)).

**Figure 8:**
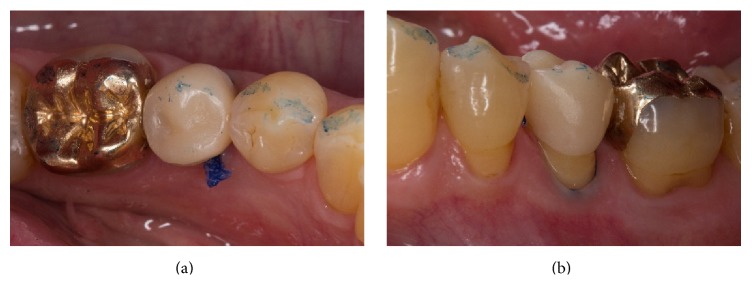
Try-in of the prepolished partial crown.

The intraoral try-in of the restoration including internal adjustment and the adjustment of the proximal contacts were the next steps. After the correct fit of the restoration was achieved, the patient was asked to bite down very carefully. Then, the occlusal contacts were marked with articulation paper (Figures 8(a) and 8(b)). Selective adjustment could be performed with water-cooled fine diamond instruments (8390.314.016, Gebr. Brasseler, Lemgo, Germany). When fabricating posterior Celtra Duo crowns and partial crowns, glaze firing is recommended, as this step increases the final stability from 210 to 370 MPa, thus matching the mechanical strength reported for long-term evaluated lithium disilicate ceramics.

**Figure 9:**
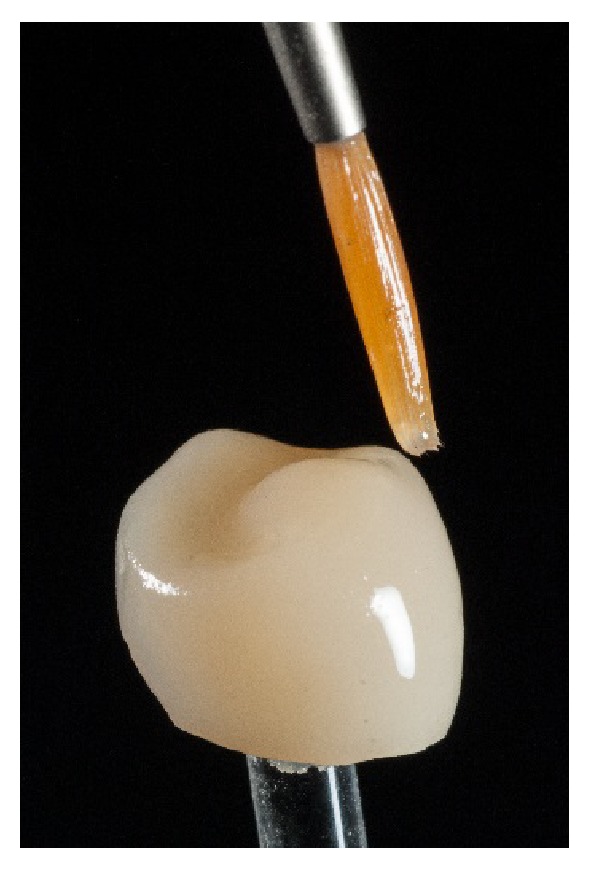
Application of the glazing material.

The restoration was cleaned by using a steam jet unit and subjected to a first glaze firing process at 820°C (heating rate 55°C/min, hold time 1 : 30 min) that was combined with an individual staining of the restoration (Celtra Universal Stain & Glaze, Dentsply DeTrey, Konstanz, Germany). It is necessary to coat the entire surface with the glazing material to obtain a uniform glossy finish (Figure 9).

**Figure 10:**
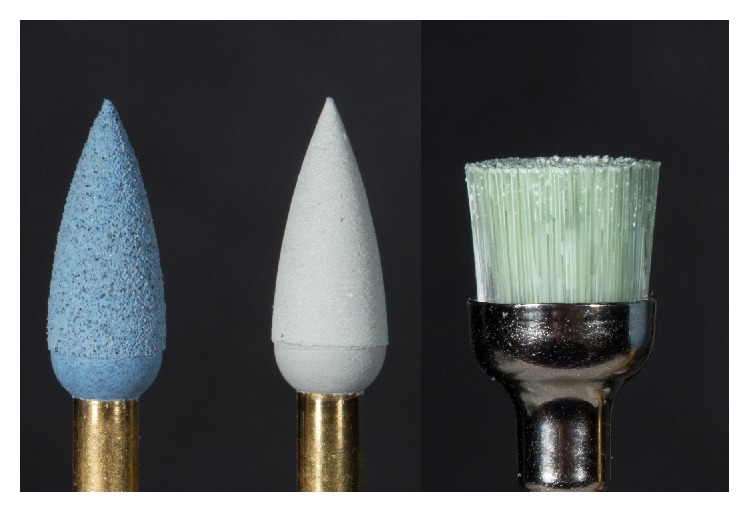
Instruments and material for the mirror finish of the restoration.

In the present case, an additional glaze firing was performed at 770°C (heating rate 55°C/min, hold time 1 : 30 min) to accentuate the shade. The restoration was then mirror-finished with diamond-impregnated polyurethane instruments (94020F.204.040. Gebr. Brasseler, Lemgo, Germany) at moderate speed (not exceeding 8,000 rpm) and using a diamond polishing paste (Direct Dia Paste, Shofu Dental, Ratingen, Germany) (Figure 10).

One hour after impression taking, the restoration was ready for a final esthetic try-in with a try-in gel (Calibra Try-In Paste, Dentsply DeTrey, Konstanz, Germany) to verify the fitting accuracy and the correct match of shade.

Only minor occlusal adjustments of the proximal contacts with diamond-impregnated polyurethane instruments were needed at this stage. The adjusted areas were repolished using a diamond polishing paste (e.g., Direct Dia Paste, Shofu Dental, Ratingen, Germany). The paste is best applied with a nylon brush without water cooling at max. 5,000 rpm.

**Figure 11:**
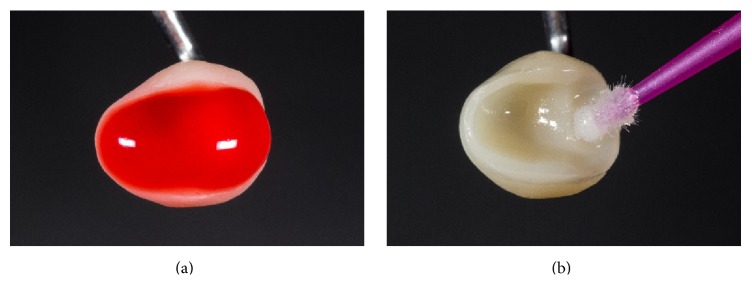
Conditioning of the ceramic restoration using 5% hydrofluoric (Vita Ceramics Etch, Vita Zahnfabrik, Bad Säckingen, Germany) acid and a silane coupling agent (Calibra Silane, Dentsply DeTrey, Konstanz, Germany).

Before adhesive luting, the surfaces of the restoration that should be bonded were conditioned with 5% hydrofluoric acid (Vita Ceramics Etch, Vita Zahnfabrik, Bad Säckingen, Germany) for 30 seconds (Figure 11(a)).

Then, all acid residues were removed by thoroughly rinsing the restorations with water (a stained etching gel allows a better control of the cleansing). The etched restorations were then dried, and a silane material (Calibra Silane, Dentsply DeTrey, Konstanz, Germany) was applied (residence time: 1 minute) (Figure 11(b)).

After cleaning the tooth with pumice and a chlorhexidine solution, it was isolated with rubber dam. 37% phosphoric acid was applied for 30 seconds to condition the enamel, while the exposed dentine was etched for 15 seconds. However, adjacent teeth should be protected with cellophane matrices before applying the etching gel. In case of unintentional conditioning of the proximal surfaces of adjacent teeth, the cement excess on these surfaces will affect further processing.

**Figure 12:**
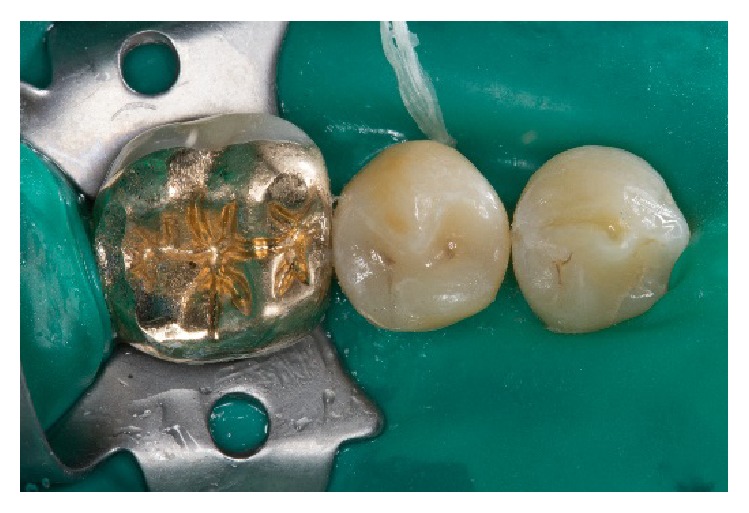
Adhesive cementation of the restoration after rubber dam application.

**Figure 13:**
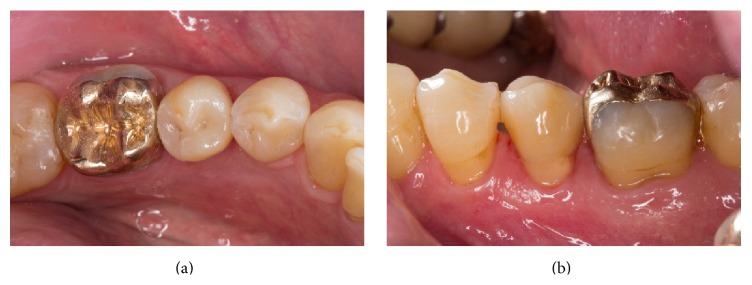
Clinical situation two days after adhesive cementation.

A dual-curing one-step adhesive (XP Bond & Self-Curing Activator, Dentsply DeTrey, Konstanz, Germany) was applied on teeth and restoration surfaces and air-thinned. A thin layer of a dual-curing transparent resin cement (Calibra automix transparent, Dentsply DeTrey, Konstanz, Germany) was applied to the preparation immediately; the restoration was then seated. The resin cement was precured for 3–5 seconds on both the lingual and the buccal side. Excess cement in the proximal areas was removed with an explorer and dental floss. The luting agent was now finally light-cured for 40 seconds on each side of the restoration (buccal/occlusal/lingual) (Figure 12). Now, the occlusal contacts were checked and adjusted where necessary. The margins were finished and polished with fine-grit size diamond instruments and the already mentioned 2-stage diamond-impregnated polishers (Figures 13(a) and 13(b)). Two weeks after cementation, the patient was reexamined and reported no postoperative sensitivity. The restorations had a good shade adaptation and postoperative tissues were healthy.

## 3. Discussion

Since the 1980s, computer-aided design and computer-aided manufacturing (CAD/CAM) have been employed in the chairside fabrication of all-ceramic restorations, especially for inlays and onlays [1, 2].

The launch of new intraoral scanning devices, as well as reliable new high-strength ceramic materials, has likewise been observed by dentists and dental technicians [4, 8, 11]. Several publications have indicated that digital techniques can replace conventional workflows for at least single-tooth restorations and short-span fixed partial dentures (FPDs) [3–7]. As one major advantage of the digital workflow, the time needed for occlusal and internal adjustments is shorter than for restorations fabricated in the conventional workflow [1, 3, 4]. This is supported by the clinical experience documented in the present case report, with only minor adjustments of the proximal and occlusal contacts which took less than 3 minutes.

Four further developments are crucial for the chairside fabrication process, as follows.

### 3.1. Intraoral Scanner

Earlier scanning systems required the time-consuming application of a scanning powder. With the introduction of powder-free scanning systems like the one applied in the present case report, the scanning process is simplified and shortened [2–4]. This is a very important factor in reducing the total fabrication time in a chairside process [4].

### 3.2. Automated Design

A high level of accuracy is needed in the design process to exactly reproduce internal as well as occlusal and proximal surfaces to enable a model-free production of an all-ceramic restoration [1, 3]. This is a precondition to avoid time-consuming intraoral adjustments or, in the worst case scenario, a remake of the restoration. With the current version of the CEREC software (4.xx), the design process has become simpler and more intuitive [2–4]. Especially for single-tooth restorations, only minor corrections by the operator are needed to modify the design templates of the software and to reach a design that meets the clinical needs regarding anatomic shape, occlusal contacts, and fitting accuracy [3]. The required time for the design of a single-tooth restoration is less than 5 minutes in the majority of cases.

### 3.3. Reduced Fabrication Time

An essential requirement for the successful integration of chairside procedures is to shorten the fabrication time as much as possible [1, 3]. Apart from a simple intraoral scanning procedure and an automated design, this is provided by the time-effective milling of the ceramic material [11]. With the material used in the present case report, a partial crown is milled within 12–14 minutes using the CEREC MXCL milling unit. Compared to other materials offering a comparable fracture strength, no crystallization firing is needed, as the final strength can be achieved with a glaze firing [11, 12]. This reduces the fabrication time to approximately 30–40 minutes [9, 11].

### 3.4. Innovative Materials

Due to their combination of strength and translucency, ZLS-ceramics offer ideal preconditions for the fabrication of monolithic restorations that are only characterized by staining [8, 11]. First in vitro studies comparing the translucency of various ceramic materials revealed a higher translucency of the ZLS-ceramic Celtra Duo compared to IPS e.max CAD in the polished state (38% versus 34%) [14]. The pronounced translucency of the material increases the so-called chameleon effect and improves the shade adaption. This effect is supported by the findings of the present clinical case report. The omission of ceramic veneering eliminates the risk of veneering ceramic fractures [2, 12]. Due to its special microstructure, this material group is easy to polish [11, 12]. All these material properties are of clinical benefit for chairside restorations. Nevertheless, at this time the material is available only in monochromatic CAD/CAM blocks, covering in parts the Vitapan classical shade range (A1–A3.5 and B2). For crowns and partial crowns, the generation of a tooth-like gradient in the shades from the cervical to the incisal area requires the use of stains in an additional firing cycle. Apart from enlarging the range of monochromatic blocks, covering the complete Vitapan shades (A–D), the fabrication of polychromatic CAD/CAM blocks with an opaque core surrounded by a translucent layer could be a meaningful innovation. This could reduce the necessity of a separate staining procedure as well as the fabrication time.

In the present study, the ZLS-ceramic was conditioned by an etching process with 5% hydrofluoric acid for 30 seconds. Based on the findings of an in vitro investigation, this pretreatment ensures a bonding strength comparable to the well-known lithium disilicate ceramics in combination with dual-curing composite cements and self-adhesive cements [16]. An alternative pretreatment by air-abrasion with 50 *μ*m aluminous oxide cannot be recommended, as this procedure only leads to 50% of the bonding strength determined for a pretreatment with hydrofluoric acid and silane application [16].

Nevertheless, it is a limitation of these new materials that data from clinical studies are missing. Moreover, financial investments for intraoral scanners, CAD software, and the milling unit are high in comparison with conventional workflows, despite considerable progress in the further development of digital workflows [3].

## 4. Conclusions

ZLS-ceramics offer a good combination of high strength and outstanding optical properties. As the materials can be milled in their finally crystallized state, they are interesting for the time-saving chairside fabrication of monolithic restorations in load-bearing areas that require a fracture strength of >350 MPa (partial crowns and full crowns). However when using ZLS-ceramics, the material specific processing instructions should be strictly observed, even though ZLS-ceramics show a positive combination of properties that were confirmed in several laboratory studies. This is especially important regarding the necessary minimum wall thickness and required adhesive luting. Results from additional clinical studies are required to validate the positive results from these initial clinical experiences.
